# Impacts of the zero mark-up drug policy on hospitalization expenses of COPD inpatients in Sichuan province, western China: an interrupted time series analysis

**DOI:** 10.1186/s12913-020-05378-0

**Published:** 2020-06-08

**Authors:** Junman Wang, Peiyi Li, Jin Wen

**Affiliations:** Institute of Hospital Management, West China Hospital, Sichuan University, Guo Xue Xiang 37, Chengdu, 610041 People’s Republic of China

**Keywords:** Zero mark-up drug policy, Hospitalization expenses, Drug expenses, Interrupted time series, Chronic obstructive pulmonary disease

## Abstract

**Background:**

Since 1950, the hospitals had been permitted to take a 15% mark-up of drug purchase price to remedy the loss of public hospitals and doctors’ salaries in China due to tight government budget. This policy resulted in an increasing over-prescriptions which increased burden for patients eventually. The soaring medical expenditures prompted Chinese government to launch the zero mark-up drug policy (ZMDP) in 2009, which aims to eliminate physicians’ financial incentives and lighten patients’ economic burden through cancelling the 15% mark-up. The purpose of this study is to assess the impacts of the ZMDP on hospitalization expenses for inpatients with chronic obstructive pulmonary disease (COPD) in western China.

**Method:**

An interrupted time series was used to assess the impact of the ZMDP in 25 tertiary hospitals of Sichuan province, in which the policy was implemented in 2017. Monthly average total hospitalization expenses including drug expenses, medical service expenses and diagnosis expenses of COPD inpatients were analyzed with segmented regression model developed from January 2015 to June 2018.

**Results:**

After the intervention of the ZMDP, the total hospitalization expenses of COPD patients significantly decreased immediately by 1022.06 CNY (*P* = .011). The post-policy long-term trend was decreasing by 125.32 CNY (*P* < .001) per month compared to the pre-policy period. The drug expenses kept downward trend both before and after the policy implementation. It had decreased by 46.42 CNY (*P* < .001) per month on average before the policy implementation and then dropped 1073.58 CNY (*P* < .001) immediately after the policy was implemented. Meanwhile, the medical service expenses had an increasing baseline trend of 14.93 CNY (*P* < .001) per month before the policy intervention, but it increased 197.75 CNY immediately after the policy was implemented (*P* = .011). The pre-policy period long-term trend of diagnosis expenses had increased by 25.78 CNY (*P* < .001) per month and decreased immediately by 310.78 CNY (*P* = .010). The post-policy trend was decreasing by 35.60 CNY (*P* = .001) per month compared to the pre-policy period.

**Conclusion:**

Our study suggested that the ZMDP have been an effective intervention to curb the increase of hospitalization expenses for inpatients with COPD, especially the drug expenses in western region of China.

## Background

Public hospitals, as non-profit medical institutions, are the main providers of healthcare services in China. In the era of planned economy, every Chinese citizen could enjoy free medical services, and the majority income of hospitals and medics was supported by the financial subsidies from the government [1]. However, the Chinese government could not afford the large healthcare inputs in a long period, the subsidies had fallen to 11% from 60% [2]. To remedy the loss in incomes of health services providers, a 15% makeup on the price of pharmaceutical products (except Chinese herbal medicine) had been allowed since 1950 [3]. The nationwide implementation of the policy enabled the medical institutions to maintain normal operation by the revenue of drugs even if insufficient government financial resources. In 1978, the Third Plenary Session of the Eleventh Central Committee inaugurated a new era of reform and opening-up, in which the government decided to loosen their control over the medical institutions and give part of their profits back to hospitals themselves so that the hospitals could organize their medical activities according to the market demand [4]. Therefore, hospitals gradually turned to be market-oriented and started to encourage their physicians to prescribe superfluous drugs with the power of self-management for their personal income was linked with drug profits. Thus, the phenomenon of over-prescriptions and high economic burden for patients gradually appeared [5]. Extremely high drug expenses have become a serious social problem in China, and seriously affected the relationship between physicians and patients [6].

In view of this, the Chinese government issued the zero mark-up drug policy (ZMDP), which proposed to eradicate the 15% profit margin for drug sales in 2009. The implementation of this policy was expected to mitigating the medical misconduct of over-prescription driving by financial incentives and finally alleviate the economic burden of the public [7]. The ZMDP was executed gradually in different regions in China, with local Ministry of Health increased subsidies to hospitals to compensate the loss of incomes.

All public hospitals in Sichuan province have removed the drug mark-up on January 1, 2017. The income reduced due to the elimination of drug markup will be compensated through various adjustments. 70% of them was be compensated via increasing the price of medical services that reflecting value of labor, technical difficulty and risk degree of treatment, and other 20% was offset through local financial input, and 10% was be compensated by hospitals themselves through cost reduction and other measures [8]. Moreover, the adjusted medical services shall be reimbursed from medical insurance, ensuring that the burden of medical expenses on patients would be reduced [9].

However, it’s need noted that the adjustments varies in different provinces which may resulted various impacts of the ZMDP. It was reported that the average medical expenses per outpatient and per inpatient decreased 25.93 and 25.22% respectively in 2011 compared with 2010 by a study investigated 60 primary medical institutions in Shandong and Hebei provinces [10]. Studies conducted by Yang et al. and Zhou et al. got similar conclusions in Shaanxi province [11, 12]. Yi et al. have found that there are large reductions in drug revenue after the implementation of this policy [13]. Also an increasing trend by 1.31% of hospitalization expenses per capita was found in Shanwei city by researchers Chen [14].

In addition, there is no study had discussed the impact of the ZMDP on hospitalization expenditures of a specific disease. Chronic obstructive pulmonary disease (COPD) is one of the main contributors to the global burden of disease, especially in China which ranks the 4th leading cause of disability adjusted life years (DALYs) [15]. In Sichuan, the number of deaths of COPD was 140,858 in 1990 and 13,478 in 2013, compare with the number of cases of COPD was 3,087,656 in 1990 and 3,469,142 [16]. Worsely, COPD puts enormous financial pressure on governments. The annual cost of COPD in Europe is 38.6 billion euros (303 billion CNY) [17] and 49.5 billion dollars (350 billion CNY) in the United States [18]. In Singapore, hospitalization accounting for 73% of the total COPD burden, or an average of 7.2 million dollars (50.8 million CNY) per year [19]. In 2013–2016, the hospitalization cost of COPD patients in Lanzhou was 8134.11 CNY, and the median value was 6794.52 CNY [20]. The patient’s economic burden of disease was excessive. Evidence from the investigation of six large cities in China shows that the total expenses of one COPD patient account for 40% of the total income of their family. Additional, drug expenses among the total medical expenses of COPD accounted for the major priority of total medical costs [21]. Therefore, in this paper, interrupted time series (ITS) was used to explore whether the ZMDP can reduce the expenses of inpatients continuously and effectively in Sichuan province, providing strong evidences for the effectiveness of the policy.

## Methods

### Setting

The Sichuan province is an agricultural and economically developing province that located in southwestern China with complex and diverse topography. The total area of Sichuan is 486,000 km^2^ and the resident population is 83.41 million in 2018 [22]. The Health Commission of Sichuan Province selected 25 tertiary hospitals in Sichuan province (all of them are tertiary hospitals) which were convinced to be the representatives of medical institutions in Western China. This study analyzed the average hospitalization expenses of COPD inpatients in these hospitals from January 2015 to June 2018 for a total of 42 months. This study was approved by Ethics Committee of West China Hospital of Sichuan University.

### Outcome variables

The hospitalization expenses mainly include drug expenses, medical service expenses and diagnosis expenses. The data used in this study came from electronic medical records at the 25 hospitals. Inpatients who hospitalized less than 2 days or more than 60 day and who didn’t accept diagnosis and treatment were excluded. Stata (Version 15; Stata Corporation College Station, TX, USA) was employed in this research to conduct the ITS statistical analysis with *P* < .05 was considered statistically significant. All the expenses have been adjusted by inflation rate to minimize errors.

### Statistical analysis

The ITS analysis is considered the strongest quasi-experimental research design. It is best understood as a simple but powerful tool used for evaluating the impact of a policy change or quality improvement program. In the simplest case, divide the time series into 2 segments. The first segment comprises rates of the event before the intervention or policy, and the second segment is the rates after the intervention. “Segmented regression” is used to measure statistically the changes in level and slope in the post-intervention period compared to the pre-intervention period. In other words, segmented regression is used to measure immediate changes in the rate of the outcome as well as changes in the trend [23].

Segmented regression models fit a least squares regression line to each segment of the independent variable, time, and thus assume a linear relationship between time and the outcome within each segment [24]. The following multivariable regression model was specified to estimate the level and trend in various costs for patients with COPD:

$$ {Y}_t={\beta}_0+{\beta}_1\times \kern0.5em time+{\beta}_2\times \kern0.5em intervention\kern0.5em +{\beta}_3\times \kern0.5em time\_\mathrm{after}\_\mathrm{intervention}\kern0.5em +\kern0.5em \upvarepsilon $$

*Y*_*t*_ is the expenses in month t; the variable time is a continuous variable indicating time in months at time t from the start of the observation period; intervention is an indicator variable for time t occurring before (0) or after (1) the policy, which was implemented at month 25 in the series; time after intervention is a continuous variable coded 0 before the policy is implemented, then sequentially numbers time periods after implementation [24].. *β*_0_ is the average cost level at the start time; *β*_1_ is the trend of monthly average cost before the implementation of the policy; *β*_2_ estimates the level change in the cost immediately after the intervention from the end of the preceding segment; and *β*_3_ estimates the change in the long-term trend in the outcome after the implementation of the policy, compared with the trend before the policy. ε is the error term at time t representing the random variability not explained by the model. We used the Durbin-Watson test to assess the existence of auto-correlations.

## Results

In our study, 64,546 inpatients with COPD across 42 months were enrolled. The detailed data of expenses from January 2015 to June 2018 are given in Additional file 1. We can find that the average total hospitalization expenses before the policy intervention was 16,926.87 CNY and it was higher than the average total hospitalization expenses which was 14,405.53 CNY after the policy intervention.

### Model 1: Total hospitalization expenses as the dependent variable

**Fig. 1:**
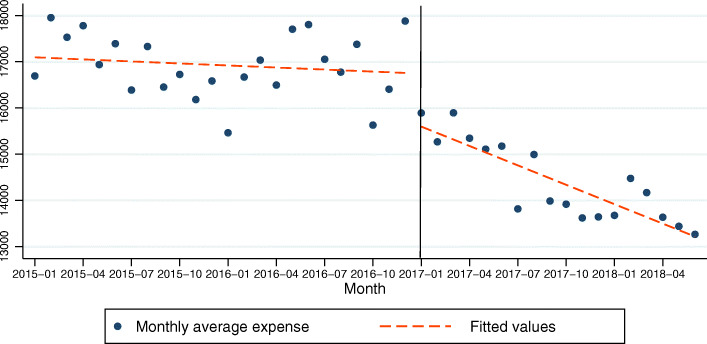
Trend in the monthly average total hospitalization expense (CNY) for 25 tertiary hospitals in Sichuan, January 2015–June 2018. The vertical line shows the time when the ZMDP was launched

In the model for total hospitalization expenses of COPD patients, the growth before the policy was positive but almost flat, not showing significant difference with respect to the pre-policy period. The intercept was 17,110.64 at the time zero and this was statistically significant (*P* < .001). The *β*_1_ was − 14.70 showing no statistical significance (*P* = .414). This indicates that there was no change in the expenses per month before the policy. The *β*_2_ was − 1022.06 and this was statistically significant (*P* = .011). This indicates that after the policy intervention, the total hospitalization expenses of COPD patients decreased immediately in level by 1022.26 CNY. The *β*_3_ was − 125.32 and this was statistically significant (*P* < .001). This indicates that the post-policy long-term trend began to be negative which was decreasing by 125.32 CNY compared to the pre-policy period (Table 1, Fig. 1). R-squared = .845. The adjusted R square was .832. The Durbin-Watson test was 1.757.

**Table 1:** Regression coefficients, standard errors, and *P*-values from the multiple regression analysis type of segmented regression analysis of interrupted time series for models 1(total hospitalization expenses as the dependent variable)

	coefficients	robust std. error	t	sig	95% Conf. Interval	
*β*_1_	−14.70	17.79	−0.83	0.414	−50.71349	21.31188
*β*_2_	−1022.06	380.82	−2.68	0.011	− 1792.993	− 251.1269
*β*_3_	−125.32	32.67	−3.84	0.000	− 191.4686	− 59.17733
*β*_0_	17,110.64	254.19	67.32	0.000	16,596.06	17,625.21

### Model 2: medical service expenses of COPD patients as the dependent variable

**Fig. 2:**
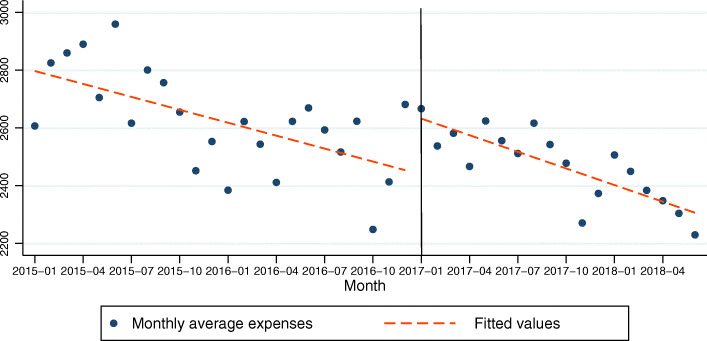
Trend in the monthly average medical service expense (CNY) for 25 tertiary hospitals in Sichuan, January 2015–June 2018. The vertical line shows the time when the ZMDP was launched

In the model for medical service expenses of COPD patients, the constant was 2811.98 and this was statistically significant (*P* < .001). The *β*_1_ was −14.93 this was statistically significant (*P* < .001). This indicates that there was a statistically significant decreasing baseline trend in medical service expenses of 14.93 CNY per month before the policy intervention. The *β*_2_ was 197.75 and that was significant (*P* = .011). This indicates that after the policy intervention, the medical service expense of COPD patients increased by 197.75 CNY immediately and that was statistically significant. The *β*_3_ was − 4.25and that was not significant (*P* = .507). This indicates that the decrease in trend in the expenses after the policy compared with the trend before the policy was not significant (Table 2, Fig. 2). The R-squared = .568. The adjusted R square was .533. The Durbin-Watson test was 1.890.

**Table 2:** Regression coefficients, standard errors, and *P*-values from the multiple regression analysis type of segmented regression analysis of interrupted time series for models 2(medical service expenses as the dependent variable)

	coefficients	robust std. error	t	sig	95% Conf. Interval	
*β*_1_	−14.93	3.46	− 4.32	0.000	− 21.92152	−7.92853
*β*_2_	197.75	73.99	2.67	0.011	47.97305	347.5245
*β*_3_	−4.25	6.35	−0.67	0.507	−17.1051	8.596238
*β*_0_	2811.98	49.38	56.94	0.000	2712.011	2911.952

### Model 3: diagnosis expenses of COPD patients as the dependent variable

**Fig. 3:**
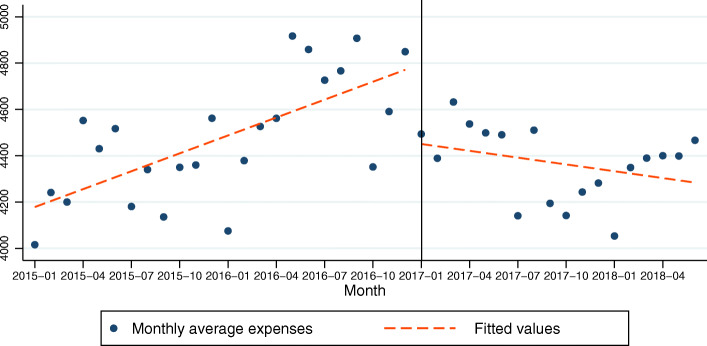
Trend in the monthly average diagnosis expense (CNY) for 25 tertiary hospitals in Sichuan, January 2015–June 2018. The vertical line shows the time when the ZMDP was launched

In the model for diagnosis expenses of COPD patients, the constant was 4152.46 at time zero, and this was statistically significant (P < .001). The *β*_1_ was 25.78 and this was statistically significant (*P* < .001). This indicates that there was a statistically significant increasing baseline trend in the expenses of 25.78 CNY per month before the policy intervention. After the policy intervention, the expense decreased immediately by 310.78 CNY (*β*_2_ = − 310.78, *P* = .010). The *β*_3_ was − 35.60 and that was significant (*P* = .001). This indicates that compared with the trend of diagnosis expenses before the policy intervention, the trend of diagnosis expenses after the policy intervention was dropped by 35.60 CNY. That is to say, after the policy intervention, the diagnosis expenses of COPD patients began to be negative and dropped by 9.82 CNY (*β*_1_ + *β*_3_) per month (Table 3, Fig. 3). The R-squared = .425. The adjusted R square was .380. The Durbin-Watson test was 1.587.

**Table 3:** Regression coefficients, standard errors, and *P*-values from the multiple regression analysis type of segmented regression analysis of interrupted time series for models 3 (diagnosis expenses as the dependent variable)

	coefficients	robust std. error	t	sig	95% Conf. Interval	
*β*_1_	25.78	5.36	4.81	0.000	14.92298	36.64426
*β*_2_	−310.78	114.85	− 2.71	0.010	− 543.2738	−78.28034
*β*_3_	−35.60	9.85	−3.61	0.001	− 55.54324	−15.64707
*β*_0_	4152.46	76.66	54.17	0.000	3997.279	4307.648

### Model 4: drug expenses of COPD patients as the dependent variable

**Fig. 4:**
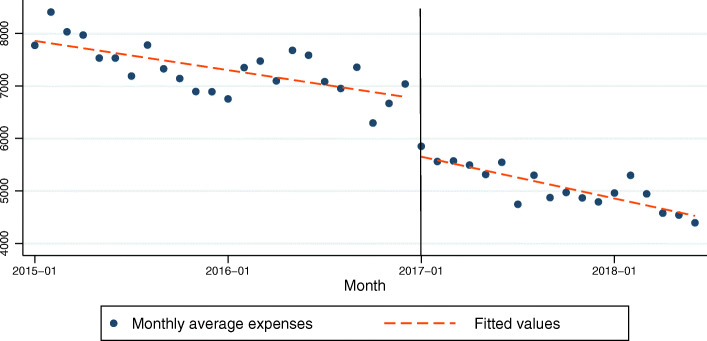
Trend in the monthly average drug expense (CNY) for 25 tertiary hospitals in Sichuan, January 2015–June 2018. The vertical line shows the time when the ZMDP was launched

In the model for drug expenses of COPD patients, the constant was 7905.49 at the time zero and this was statistically significant (*P* < .001). The *β*_1_ was − 46.42 and that was statistically significant (*P* < .001). This indicates that there was a significant change in the drug expenses before the policy was implemented. The drug expenses dropped by 46.42 CNY per month on average. The *β*_2_ was − 1073.58 and this was statistically significant (P < .001). This indicates a significant level decrease in the drug expenses after the policy. After the policy intervention, the drug expense dropped 1073.58 CNY immediately. The *β*_3_ was − 19.88 but this was not significant (*P* = .248). This indicates that the change in the trend in the drug expenses after the policy was implemented with the trend before the policy was implemented was not significant (Table 4, Fig. 4). The R-squared = .938. The adjusted R square was .933. The Durbin-Watson test was 1.571.

**Table 4:** Regression coefficients, standard errors, and *P*-values from the multiple regression analysis type of segmented regression analysis of interrupted time series for models 2(drug expenses as the dependent variable)

	coefficients	robust std. error	t	sig	95% Conf. Interval	
*β*_1_	−46.42	9.23	−5.03	0.000	−65.10885	−27.73629
*β*_2_	−1073.58	197.60	−5.43	0.000	− 1473.601	− 673.5567
*β*_3_	−19.88	16.95	−1.17	0.248	−54.20093	14.44242
*β*_0_	7905.49	131.89	59.94	0.000	7638.487	8172.492

## Discussion

This is the first study that assessed the impact of the ZMDP in Sichuan province by ITS. We chose the COPD population which is one of the major chronic diseases affecting human health [15]. Even if no control group is set, the ITS model can control and eliminate the influence of long-term trend changes of the time series before the intervention on the results through the analysis of multiple observation time points data, so as to correctly evaluate the real effect of the intervention on the results [25].

After the ZMDP intervention, the total hospitalization expenses dropped significantly. Subsequently, the total hospitalization expense began to show a downward trend. It is worth noticing that the drug expense, which is the mainly part of total hospitalization expenses, dropped significantly and immediately after the policy intervention. The diagnosis expense showed a rising trend before the intervention, but there was a decline immediately after the policy and the post-policy trend began to be negative. However, the medical service expense increased immediately after the policy intervention.

In line with our findings, previous studies demonstrated that the ZMDP can alleviate the economic burden of patients. One study by Fu and Yang showed a decrease in drug and diagnosis, but an increase in medical service expenses after the policy intervention which was consistent with our study [26]. Another study showed that without the mark-up of drugs, the expense of cesarean section in primary hospitals had been decreased and the phenomenon of over-prescription had been curbed [27]. However, these studies did not use ITS to access the impact of the ZMDP on medical expenses so that they might not able to measure the immediate changes in the expenses as well as changes in the trend in post-policy period.

China’s health care reform has been expected to alleviate the economic burden of patients and get better medical services [28–31]. It is clear that the ZMDP has significant impact on hospitalization expenses of COPD patients especially the drug expenses. Meanwhile, the increase of medical service expenses after the policy intervention may due to the reform of the medical and health care system [32, 33]. In 2017, the Chinese government call upon that Minstry of Health must accelerate the establishment of a timely and flexible mechanism for dynamic price adjustment, and optimize the price of medical service, especially the price that reflects the value of medical personnel’s technical labor by standardizing medical treatment behavior and reducing the prices of large medical equipment inspection, treatment and inspection [34]. It hadn’t brought any economic burden to inpatients since the total hospitalization expenses had kept decreasing; in contrast, it had encouraged the medical staff to provide better medical service [26]. The diagnosis expenses after the intervention showed a downward trend which was beyond our expectation. Because some hospitals may develop adaptive strategies to address reduction in revenue by providing financial incentives for doctors to excessive diagnosis [13]. The government should take appropriate measures to prevent it.

This study suffers from several limitations. Firstly, the data of other possible explanatory variables such as age, status of smoking, treatments and drug use were not able to obtain. Secondly, we merely collect tertiary hospitals in the survey, which thereby limited the possibilities for investigation of other secondary hospitals and primary healthcare centers to the impacts of ZMDP. Finally, we didn’t consider the mode of payment of inpatients [35]. For example, if the health insurance of patients is different, the hospitalization expenses after reimbursement may be different. Therefore, population-based economic evaluation studies of a wider range should be conducted on the effectiveness of COPD drug treatment in the future [36]. In addition, more similar studies for other disease and other region are warranted.

## Conclusion

In conclusion, the ZMDP in Sichuan province had a successful impact on hospitalization expenses for inpatients with COPD. Especially the drug expenses decreased dramatically. To some extent, the ZMDP appears to have been an effective public health intervention to alleviate patients’ economic burden. The results of this study on policy interventions may be helpful for other under-developed countries that intend to reduce their citizens’ medical expenses.

## Supplementary information

**Additional file 1.** Changes in total hospitalization expenses, medical service expenses, diagnosis expenses, and drug expenses of COPD patients in 25 tertiary hospitals of Sichuan province from January 2015 to June 2018.

## Data Availability

The datasets generated or analysed during the current study are not publicly available due confidentiality policies but are available from the corresponding author on reasonable request.

## Abbreviations

ZMDP: Zero mark-up drug policy
COPD: Chronic obstructive pulmonary disease
ITS: Interrupted time series
