# Supplementary material for: Impacts of the zero mark-up drug policy on hospitalization expenses of COPD inpatients in Sichuan province, western China: an interrupted time series analysis
Source: BMC Health Serv Res. 2020 Jun 8;20:519. doi: 10.1186/s12913-020-05378-0 (PMC7282107; doi:10.1186/s12913-020-05378-0)
Supplement: Supplementary file 1 — Additional file 1. Changes in total hospitalization expenses, medical service expenses, diagnosis expenses, and drug expenses of COPD patients in 25 tertiary hospitals of Sichuan province from January 2015 to June 2018. [file 12913_2020_5378_MOESM1_ESM.pdf]

Additional file 1: Changes in total hospitalization expenses, medical service expenses, diagnosis expenses, and drug expenses of COPD patients in 25 tertiary hospitals of Sichuan province from January 2015 to June 2018

Date	Total hospitalization expenses(CNY)	Medical service expenses(CNY)	Diagnosis expenses (CNY)	Drug expenses (CNY)	Time	Intervention	Time after intervention
15-Jan	16692.72	2606.83	4015.56	7774.40	1	0	0
15-Feb	17954.83	2825.10	4240.99	8409.13	2	0	0
15-Mar	17530.23	2859.85	4199.89	8032.23	3	0	0
15-Apr	17780.46	2889.98	4552.24	7970.62	4	0	0
15-May	16938.41	2705.11	4430.30	7532.36	5	0	0
15-Jun	17390.75	2959.39	4516.93	7532.02	6	0	0
15-Jul	16387.42	2616.67	4180.47	7189.32	7	0	0
15-Aug	17330.26	2800.70	4340.18	7780.31	8	0	0
15-Sep	16451.50	2756.75	4135.56	7327.22	9	0	0
15-Oct	16726.10	2654.96	4350.02	7142.27	10	0	0
15-Nov	16181.33	2451.82	4359.82	6893.67	11	0	0
15-Dec	16584.68	2552.95	4561.93	6889.52	12	0	0
16-Jan	15462.61	2384.53	4075.12	6753.05	13	0	0
16-Feb	16668.71	2622.11	4378.77	7349.85	14	0	0
16-Mar	17035.86	2543.68	4526.47	7475.50	15	0	0
16-Apr	16495.40	2411.47	4561.67	7097.33	16	0	0

16-May	17705.21	2622.76	4917.20	7678.71	17	0	0
16-Jun	17804.40	2669.27	4859.09	7585.94	18	0	0
16-Jul	17053.57	2593.12	4726.37	7082.91	19	0	0
16-Aug	16776.20	2516.68	4766.43	6951.76	20	0	0
16-Sep	17378.06	2623.19	4907.39	7356.97	21	0	0
16-Oct	15628.99	2248.61	4351.56	6294.05	22	0	0
16-Nov	16406.18	2413.19	4590.76	6667.91	23	0	0
16-Dec	17881.12	2681.32	4849.48	7037.93	24	0	0
17-Jan	15891.12	2666.70	4493.84	5849.84	25	1	25
17-Feb	15266.32	2537.62	4389.03	5560.13	26	1	26
17-Mar	15894.07	2581.75	4631.93	5571.14	27	1	27
17-Apr	15345.10	2466.76	4537.17	5491.95	28	1	28
17-May	15107.32	2624.20	4498.79	5313.12	29	1	29
17-Jun	15173.73	2555.81	4490.58	5544.59	30	1	30
17-Jul	13815.15	2511.50	4140.62	4744.58	31	1	31
17-Aug	14991.78	2616.65	4510.49	5298.11	32	1	32
17-Sep	13985.50	2542.50	4194.20	4872.63	33	1	33
17-Oct	13916.87	2477.86	4141.72	4969.97	34	1	34
17-Nov	13619.27	2270.99	4243.14	4866.53	35	1	35
17-Dec	13641.78	2373.29	4282.07	4790.44	36	1	36
18-Jan	13674.50	2506.51	4053.15	4960.22	37	1	37
18-Feb	14475.12	2449.61	4349.03	5296.55	38	1	38

18-Mar	14167.41	2383.83	4389.72	4943.34	39	1	39
18-Apr	13634.21	2348.12	4400.06	4576.18	40	1	40
18-May	13436.95	2304.29	4398.89	4539.85	41	1	41
18-Jun	13263.34	2229.85	4466.67	4393.06	42	1	42
